# Benchmarking time to initiation of end-of-life homecare nursing: a population-based cancer cohort study in regions across Canada

**DOI:** 10.1186/s12904-018-0321-5

**Published:** 2018-05-04

**Authors:** Hsien Seow, Danial Qureshi, Lisa Barbera, Kim McGrail, Beverley Lawson, Fred Burge, Rinku Sutradhar

**Affiliations:** 1 Department of Health Research Methods, Evidence, and Impact, McMaster University, Hamilton, ON Canada; 2 Institute for Clinical Evaluative Sciences, Toronto, ON Canada; 3 University of Toronto, Toronto, ON Canada; 4 University of British Columbia, Vancouver, BC Canada; 5 Department of Family Medicine, Dalhousie University, Halifax, NS Canada; 6 Department of Oncology, McMaster University, Hamilton, ON Canada

**Keywords:** Cancer, Palliative care, Benchmarking, End-of-life homecare nursing

## Abstract

**Background:**

Several studies have demonstrated the benefits of early initiation of end-of-life care, particularly homecare nursing services. However, there is little research on variations in the timing of when end-of-life homecare nursing is initiated and no established benchmarks.

**Methods:**

This is a retrospective cohort study of patients with a cancer-confirmed cause of death between 2004 and 2009, from three Canadian provinces (British Columbia, Nova Scotia, and Ontario). We linked multiple administrative health databases within each province to examine homecare use in the last 6 months of life. Our primary outcome was mean time (in days) to first end-of-life homecare nursing visit, starting from 6 months before death, by region. We developed an empiric benchmark for this outcome using a funnel plot, controlling for region size.

**Results:**

Of the 28 regions, large variations in the outcome were observed, with the longest mean time (97 days) being two-fold longer than the shortest (55 days). On average, British Columbia and Nova Scotia had the first and second shortest mean times, respectively. The province of Ontario consistently had longer mean times. The empiric benchmark mean based on best-performing regions was 57 mean days.

**Conclusions:**

Significant variation exists for the time to initiation of end-of-life homecare nursing across regions. Understanding regional variation and developing an empiric benchmark for homecare nursing can support health system planners to set achievable targets for earlier initiation of end-of-life care.

## Background

Providing end-of-life care in the home is an important policy issue because it can support many patients’ preferences to die at home [1, 2], and has the potential for cost-savings by avoided hospitalizations [3–5]. In particular, homecare nursing is critical at end-of-life since it provides complex symptom management, education, and support, which can then help to avoid often unnecessary and expensive hospitalizations. Yet policymakers have little information or quality benchmarks about the delivery of end-of-life homecare nursing.

Research has shown that the use and intensity of end-of-life homecare nursing is strongly associated with a home death [6], and reduced hospitalizations near death [7]. Moreover, several studies have demonstrated that early initiation of palliative and end-of-life care in cancer patients has benefits, such as reduced symptoms, improved quality of life, and even longer survival [8–10]. Earlier initiation and more intensity of end-of-life homecare nursing specifically, has also been associated with reduced hospitalizations and hospital deaths [7, 11, 12]. Yet despite the growing evidence of the benefits of early initiation of end-of-life care by homecare nurses, little research describes the timing in when such care is initiated. This hinders health system planners from understanding regional variation in end-of-life home care access. Furthermore, there are no established benchmarks to compare or target against.

The purpose of this study is three-fold: i) to describe the regional variation in the mean time to first end-of-life homecare visit starting from 6 months before death among health regions across British Columbia (BC), Nova Scotia (NS), and Ontario (ON); ii) establish the three-province average with confidence limits for this outcome; and iii) determine a national empiric benchmark based on the best regional performers. Ultimately these data can be used by Canadian policy makers to set achievable targets and improve end-of-life home care access. The methods can be applied by other countries to determine their own benchmarks.

## Methods

### Study design

We conducted a retrospective cohort study of cancer decedents who received homecare nursing in the Canadian provinces of ON, NS and BC, which contain a total of 28 health regions. Our inclusion criteria were adult decedents (19 years or older) with a valid provincial health insurance number, who had at least one record of homecare nursing following their cancer diagnosis and within 6 months of their date of death during the study period between April 1, 2004 and March 31, 2009. Due to a time lag in capturing cancer-confirmed cause of death in cancer registries, these were the most recent data available at the time of study inception.

### Data sources

To derive our cohort, we used a unique encrypted patient identifier within each province to link with multiple administrative databases. Starting with a provincial cancer registry for cancer type, cancer diagnosis, and confirmed cause of death from cancer, we then linked with the: provincial homecare database for homecare nursing use with standard or end-of-life intent; Canadian Institute for Health Information’s Discharge Abstract Database for Charlson-Deyo score for comorbidity; and the provincial health insurance databases for demographics of age at death, sex, and postal code for both region and income quintile [13–16]. Individual level data were not merged across provinces.

### Outcome

Our main outcome was mean number of days to first end-of-life homecare nursing visit, starting from 6 months (182 days) before death. Homecare nursing intent is recorded in each provincial homecare database. Standard intent nursing is provided to patients with service goals ranging from providing “short-term care” with a predictable recovery (e.g., wound care) to “preserving the client’s level of function and autonomy” with a prognosis of very gradual decline (e.g., early onset of frailty) [17]. Whereas end-of-life nursing intent is provided to patients classified as “not responsive to curative treatment and are dying,” with service goals being “to alleviate distressing symptoms to achieve the best quality of life by providing complex support in the last stages of their illness,” and a typical prognosis of an “expected death within six months” [17]. Given this standardized homecare eligibility criteria, we aligned our outcome definition to start from 6 months from death; thus, a shorter mean time would represent earlier initiation, and the outcome has a maximum of 182 days representing death date. At each week, the number of patients receiving standard or end-of-life homecare nursing was identified. After first end-of-life homecare nursing visit, all subsequent nursing visits were considered end-of-life. Nursing visits were measured as time (hours/day) in ON, as nursing visits received in BC, and as authorized in NS over a month.

### Statistical analyses

Through the use of descriptive statistics, baseline characteristics of each population under study were compared by province. The outcome was compared across health regions through the use of a bar chart graph and a X^2^ test with *P* < .05 considered as statistically significant. The benchmark mean for the outcome of interest was determined using the pared-mean method, which derives benchmarks from the top decile of performers [18]. Five criteria are included for this method of benchmark development: 1) the level of the benchmark signifies excellence, that is, it is always better than the mean; 2) the benchmark is achievable and realistic; 3) high performers should be selected from all performers in a pre-defined way; 4) all high performers should contribute to the benchmark; and 5) high performers with a low number of cases should not excessively impact the benchmark level (but should contribute).

In order to construct benchmark levels, health regions were first ranked according to descending order of performance. Starting with the best performing health region (i.e. shortest mean time), the eligible population sizes in each region were summed sequentially until the combined population size of this subset of regions was at least 10% of the combined size of all health regions. Combining the number of patients from these best-performing regions, the benchmark mean was calculated as the weighted average of the mean time to initiation of end-of-life nursing among the best performing regions.

A funnel plot was created for the outcome, controlling for age and sex. The funnel plot displays the number that received end-of-life homecare in each region on the horizontal axis and the corresponding mean days to initiation of end-of-life homecare nursing on the vertical axis (as seen by the dots). This funnel plot also illustrates the overall mean of the outcome across all regions (as seen by the thick black line) and the expected 95% and 99.8% CIs (as seen by the curved black lines) calculated based on normal distribution control limits [19]. The benchmark mean calculated above was overlaid on the funnel plot, as indicated by the red line. Statistical analyses were performed using SAS 9.3 (SAS Institute, Cary, North Carolina, USA), R 3.0.1, and Microsoft Excel 2010.

## Results

The study identified 85,339 cancer decedents who used any homecare nursing in the last 6 months of life, of which 61,903 of those used end-of-life care nursing (73%) to form our final cohort: 28% were from BC, 65% from ON, and 7% from NS. Table 1 shows the size and demographic information for the 28 regions examined in the three provinces. The average age was 71 years old, 48% were female, and approximately 20% had a comorbidity other than cancer.

**Table 1:** Cohort demographics

Province - Region #	Any Homecare Nursing Cohort, n	End-of-Life Homecare Nursing Sub-cohort, n (% of any nursing)	Median Age (IQR)	Female (%)	Wealthiest Income Quintile (%)	Deyo-Charlson Index of 1 or greater (%)
BC - 2	7104	6447 (91%)	71 (61–79)	48%	17%	14%
BC - 4	5902	5328 (90%)	73 (63–81)	47%	21%	13%
BC - 3	4225	3663 (87%)	72 (62–80)	49%	24%	14%
BC - 5	1242	1061 (85%)	68 (59–76)	44%	20%	20%
BC - 1	1021	878 (86%)	72 (64–80)	44%	12%	16%
NS - 9	1891	1633 (86%)	71 (61–80)	45%	20%	14%
NS - 8	824	701 (85%)	71 (62–80)	48%	14%	28%
NS - 3	478	406 (85%)	73 (64–81)	47%	15%	16%
NS - 4	450	375 (83%)	72 (62–80)	48%	18%	16%
NS - 2	412	372 (90%)	71 (62–81)	43%	12%	19%
NS - 1	377	315 (84%)	73 (62-(81)	47%	29%	11%
NS - 6	337	321 (95%)	73 (63–81)	42%	17%	22%
NS - 7	246	193 (78%)	76 (62–84)	45%	19%	23%
NS - 5	238	179 (75%)	71 (62–79)	48%	13%	21%
ON - 4	8256	5742 (70%)	71 (60–79)	48%	17%	18%
ON -9	6883	4696 (68%)	70 (60–80)	49%	15%	18%
ON - 11	6048	4487 (74%)	70 (62–78)	49%	22%	16%
ON - 8	5811	3885 (67%)	71 (60–80)	48%	21%	18%
ON - 7	4557	3307 (73%)	71 (59–79)	51%	30%	18%
ON - 2	5382	2710 (50%)	70 (60–78)	48%	18%	16%
ON - 3	3227	2565 (79%)	71 (60–78)	48%	21%	16%
ON - 1	3969	2552 (64%)	70 (64–79)	48%	18%	19%
ON - 6	3762	2459 (65%)	70 (59–77)	49%	29%	16%
ON - 13	3534	2121 (60%)	70 (62–79)	46%	13%	22%
ON - 10	3184	1846 (58%)	71 (60–78)	45%	15%	16%
ON - 12	2692	1752 (65%)	72 (60–78)	46%	24%	19%
ON - 5	2287	1302 (57%)	69 (61–79)	48%	12%	17%
ON - 14	1000	607 (61%)	70 (61–77)	46%	17%	23%

**Fig. 1:**
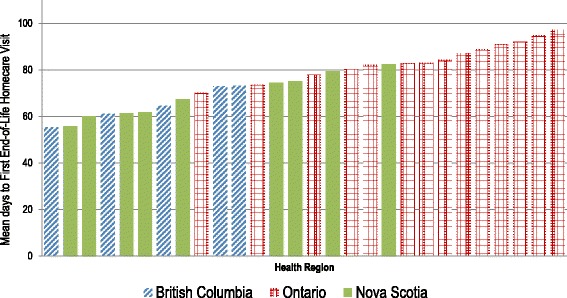
Regional variation of mean days from 6 months prior to death to first end-of-life homecare visit

Figure 1 displays the unadjusted mean number of days to initial end-of-life homecare visit (starting from 6 months before death) by the 28 regions, color-coded by province (shorter number of days to initiation equates to earlier access to end-of-life care). Large regional variations were observed. The longest mean time, 97 days, (ON- Mississauga Halton) is nearly twice as long as the shortest, 55 days (BC- Vancouver Coastal, and NS- South West). The majority of ON regions have the longest mean time to initial visit. BC and NS had the first and second shortest times, respectively.

**Fig. 2:**
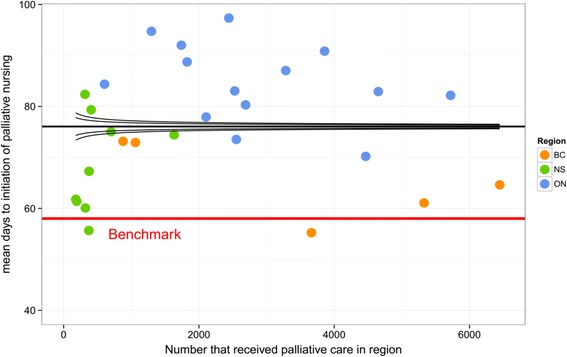
Funnel plot of mean days to initial end-of-life homecare nursing visit by region

Figure 2 displays a funnel plot for the mean days to initial end-of-life homecare nursing visit, from 6 months before death, across the 28 regions controlling for age, sex, and region size. The funnel plot includes the following information: 1) the overall mean (straight black line), 2) the expected 95% Confidence Intervals (narrows as the number that received end-of-life care in each region increases), 3) the benchmark mean (straight red line), and 4) the mean of each region (dots, color-coded according to province). The overall three-province outcome average was 76 days after the six-month point (i.e. 106 days or 3.5 months before death). The empiric benchmark value based on best-performing regions was 57 days after the six-month point (i.e. 125 days or 4.2 months before death). The best-performing regions belong to BC and NS. Most regions of ON are worse than the benchmark and overall mean. This contrasts with most regions of NS and BC, which are better than the overall mean. Regions with both small and large population sizes performed worse than the benchmark and worse than the overall indicator mean. Similarly, there were large and small regions at or near the benchmark.

## Discussion

This population-based, end-of-life cancer study displays variations in timing of end-of-life homecare nursing among 28 regions across three Canadian provinces (BC, NS, ON). While past research has demonstrated the important benefits of early end-of-life homecare, this is the first study that compares the time to initiation of end-of-life homecare visits and establishes an empiric benchmark: 57 days after the six-month point (i.e. 125 days or 4.2 months before death). We found a nearly two-fold difference in the timing of when end-of-life homecare nursing was initiated among select regions. Starting from 6 months before death, BC generally has regions with the shortest times to initiation and ON has regions with the longest times to initiation (i.e. initiated closest to death) of end-of-life homecare. Ultimately this information is useful for health system planners aiming to monitor health system performance and improve policies related to end-of-life homecare access.

Prior cancer-based randomized controlled trials demonstrating the benefits of early palliative care (i.e. at late-stage diagnosis [9] or 8–12 weeks from diagnosis [8]) were conducted in hospital-based programs: Intervention patients received early palliative care and survived for a median of 11.6 months [9] and 14 months [8], respectively. In comparison, our patients received home-based end-of-life care for a far shorter period, ranging from 125 to 83 days before death. The variation in timing may reflect the differences between palliative approach to care versus end-of-life care and/or the differences between implementing a resource-intensive home-nursing program versus a hospital program.

Past research has also used similar benchmark methods to establish quality indicators for end-of-life cancer care, such as Emergency Department visits, physician visits, Intensive Care Unit admissions, and deaths in hospital [20]. This study extends those indicators by establishing “time to initiation of end-of-life homecare nursing” as an additional quality indicator, and is related to early initiation of supportive care rather than over-aggressive acute care. In prior research, BC was identified as having the lowest hospital death rate; our study corroborates this trend as the BC regions had among the shortest mean times to first end-of-life homecare nursing. Further research is required to understand differences in provincial policies that may explain variation. Other countries could apply these methods to determine their own regional variation and benchmarks and compare against Canadian rates.

The presentation of benchmark data using a funnel plot allows health system planners to observe their performance among regions of similar, smaller, or larger sizes. It is worth noting that the two regions better than the benchmark mean are very different in size (*n* = 372 and *n* = 3663), suggesting that region size is not necessarily the most important factor in early initiation of end-of-life homecare nursing. In fact, several small, medium, and large sized regions are near the benchmark. Another advantage of this approach is that often times, benchmark values are set by larger regions or based on crude unadjusted rates. As such smaller regions find it difficult to compare themselves to larger regions. Using this approach, other regions near to or worse than the overall mean can identify a realistic comparator mean, such as a region of similar size. As a result, this supports quality improvement by setting realistic targets that regions and/or provinces can aim to achieve and may help to reduce overall variation, particularly when using recent data and monitoring over time. Moreover, health system planners can compare against better performing regions and identify what policies they have that facilitate improved access.

This study is limited by including only cancer decedents in the analysis. Using a population-based decedent cohort prevents us from examining if and when individual patients ought to have received end-of-life home care nursing [21]. However, using retrospective decedent cohorts have been shown to be an efficient way to monitor performance at a population level [22], particularly as palliative care continues to be advocated earlier in the disease trajectory [9]. The quality of end-of-life homecare nursing is not described in administrative data. Additionally, we are unable to take other factors into account that may play a role in our results. For instance, patient preferences, level of need, refusal of services, or level of caregiver support or burnout are not available in our databases. As a result, we are not able to determine appropriateness of timing at an individual-level (more clinically relevant at a patient level), but rather we can only look at variation in timing at regional population level (more relevant for health system policy planner level). Further research should explore timing of homecare services based on comprehensive patient-level factors. Strengths of the study include using a population-based cohort from three provinces, comprising approximately 54% of the population in Canada. Hence, the results possess high external validity, which likely can be generalized to the entire Canadian population. Computing benchmark values through the use of real-world data gives insight into what is required for a practical and realistic target goal. Furthermore, the methods to explore regional variation and determine benchmarks would be relevant to other countries, particularly those with publicly-funded home care systems, though the actual benchmark values might differ.

## Conclusions

In conclusion, we identified regional variation and empiric benchmarks for time to initiation of end-of-life homecare nursing using administrative health care data from three provinces. Identifying a benchmark using the best-performing regions is a beginning step. Understanding the reasons for variation, particularly provincial variation, could enable more equitable care. Exploring health system processes within best-performing regions could provide insights for other regions trying to initiate homecare services earlier. Ongoing surveillance efforts are essential to enable timely and realistic targets. National-level data sharing arrangements will allow more rapid access to regional and provincial comparisons and system performance measures.

## Abbreviations

BC: British Columbia
NS: Nova Scotia
ON: Ontario
